# Obesity, Type 2 Diabetes, and Cancer Risk

**DOI:** 10.3389/fonc.2020.615375

**Published:** 2021-02-02

**Authors:** Tiffany Scully, Abora Ettela, Derek LeRoith, Emily Jane Gallagher

**Affiliations:** ^1^Division of Endocrinology, Diabetes and Bone Disease, Icahn School of Medicine at Mount Sinai, New York City, NY, United States; ^2^Tisch Cancer Institute at Mount Sinai, Icahn School of Medicine at Mount Sinai, New York City, NY, United States

**Keywords:** obesity, Type 2 diabetes, cancer, insulin, IGF-1, lipids, leptin

## Abstract

Obesity and type 2 diabetes have both been associated with increased cancer risk and are becoming increasingly prevalent. Metabolic abnormalities such as insulin resistance and dyslipidemia are associated with both obesity and type 2 diabetes and have been implicated in the obesity-cancer relationship. Multiple mechanisms have been proposed to link obesity and diabetes with cancer progression, including an increase in insulin/IGF-1 signaling, lipid and glucose uptake and metabolism, alterations in the profile of cytokines, chemokines, and adipokines, as well as changes in the adipose tissue directly adjacent to the cancer sites. This review aims to summarize and provide an update on the epidemiological and mechanistic evidence linking obesity and type 2 diabetes with cancer, focusing on the roles of insulin, lipids, and adipose tissue.

## Introduction

**Figure 1:**
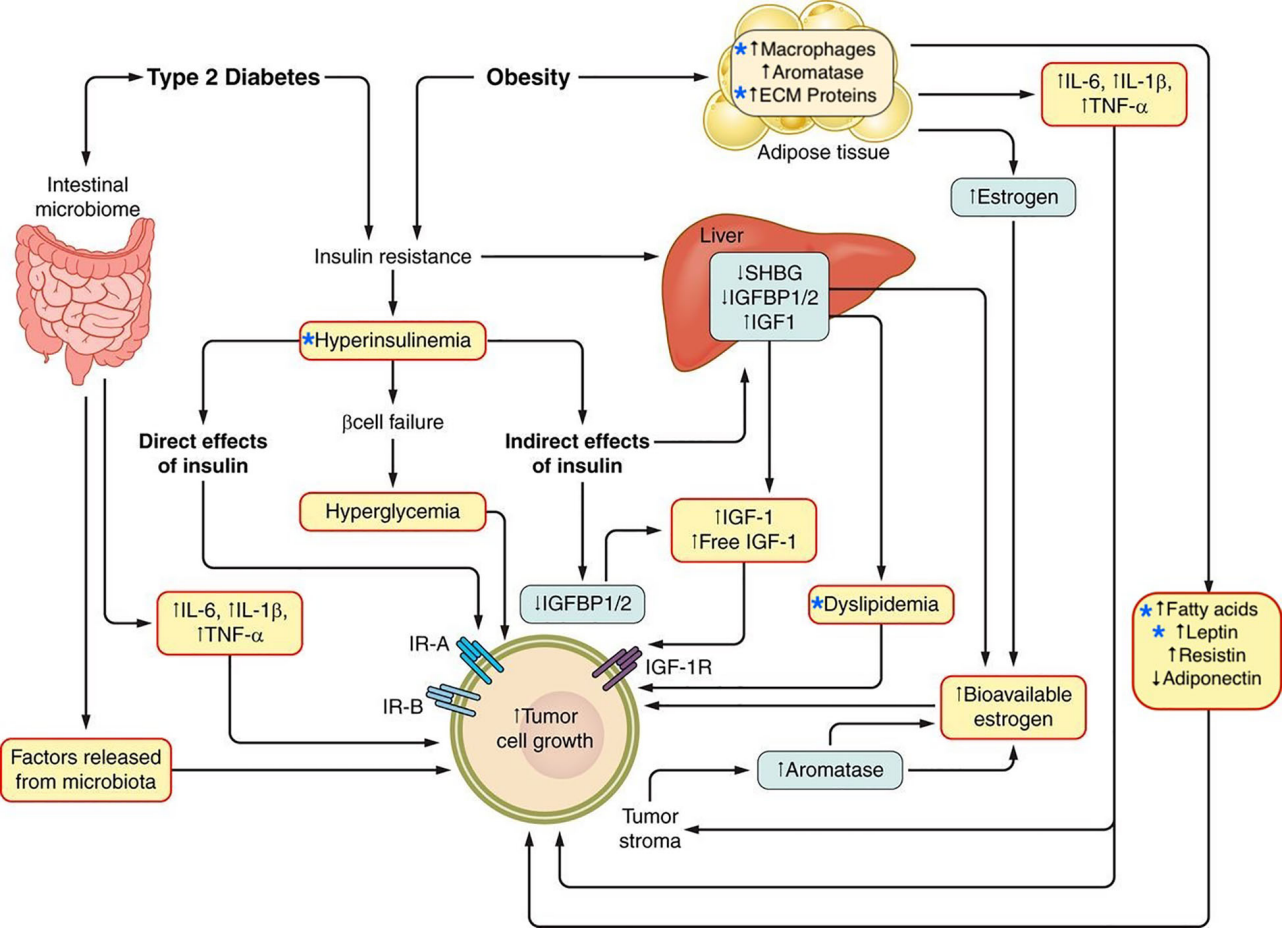
Potential mechanisms linking obesity and type 2 diabetes and cancer. The relationship between type 2 diabetes, obesity, and cancer is potentially mediated by multiple mechanisms, including metabolic conditions such as hyperinsulinemia and dyslipidemia as well as the alteration of adipose tissue which is characterized by inflammation and a tumor growth-promoting secretory profile. Stars indicate factors discussed in this review. Adapted from: Gallagher, E.J., and LeRoith, D (2015). Obesity and Diabetes: The Increased Risk of Cancer and Cancer-Related Mortality. Physiol. Rev. *95*, 727–748.

An increase in obesity has been observed in children as well as adults, in both genders, and is prevalent in both developed and developing countries (1–3). Obesity is associated with an increased risk of overall mortality (4, 5) and constitutes a risk factor for diseases such as type 2 diabetes, dyslipidemia, hypertension, fatty liver disease and cardiovascular disease. In addition, obesity has been linked to increased cancer incidence and mortality (6–8). It has been estimated that 3.6% of all of new cancer cases diagnosed worldwide in adults aged 30 years and older could be attributed to high BMI (9). An assessment of temporal trends for cancer cases in the US suggests that development of some obesity-related cancers in younger generations is becoming increasingly common (10). Internationally, an increase in obesity-related cancers in adolescents and young adults has also been noted (11), highlighting the influence of obesity on cancer risk across ages. There is therefore, much interest in understanding how obesity-associated tumor growth is mediated and might be therapeutically targeted. This review aims to provide an update on some of the mechanisms proposed to underpin the relationship between obesity and cancer (**Figure 1**), with a focus on breast cancer.

## Obesity and Type 2 Diabetes

### Obesity

Obesity has been defined as an accumulation of fat mass at levels sufficiently high to adversely influence health (12). Body mass index (BMI) is one measurement used by the World Health Organization to define obesity, and is calculated as: body weight (kg)/height (m)^2^. Overweight is considered to be a BMI of 25–29.9 kg/m^2^ and obesity, a BMI of ≥30 kg/m^2^. Of note, lower BMI cut-off points have been applied to Asian populations, due to the increased percentage of body fat in these populations, compared to non-Asian populations, for a given BMI (13, 14).

A number of studies have examined the links between cancer and obesity, defined by BMI. Large cohort studies and meta-analyses, have reported the association between obesity and cancer to be gender-, site- and menopausal status-specific (6, 7). The International Agency for Research on Cancer (IARC) and World Cancer Research Fund/American Institute for Cancer Research (WCRF/AICR) have reviewed the strength of the evidence linking obesity with specific cancer types (15–17). The IARC assessed the cancer-preventative effect of the absence of excess adiposity. Both organizations found adequate evidence supporting the association of excess body fatness with increased risk of esophageal adenocarcinoma, colon and rectal, liver, pancreatic, postmenopausal breast, endometrial, and renal cell cancer (15–18). Additional cancers for which the WCRF/AICR identified a greater risk were gastric cardia, gallbladder, ovary, mouth, pharynx and larynx, and advanced prostate cancer (17, 18).

A study assessing the effect of adolescent obesity on cancer risk and mortality later in life found that BMI at age 17 was associated with an increased overall risk of cancer in men, but not in women. Inverse relationships were reported for BMI and both breast and cervical cancers in women (19). The cancer sites most strongly connected to adolescent obesity for men in this cohort were breast, pancreas and kidney, and in women, uterus, liver, bile duct, and pancreas. Increased BMI at adolescence was also linked with a greater risk of mortality in cancer-bearing individuals (19). Several studies suggest that accumulating adiposity throughout adulthood influences cancer risk (20–23). Weight gain (≥ 0.45 kg per year) over the course of 14 years increased cancer risk by 38% compared to the maintenance of constant weight (21).

The relationship between excess adiposity and breast cancer appears to be modulated by menopausal status and by breast cancer subtype, which is clinically based on the expression of the estrogen receptor (ER), progesterone receptor (PR), and human epidermal growth factor receptor-2 (HER2). Weight gain and elevated BMI have frequently been associated with an increased risk of postmenopausal breast cancer, particularly ER-positive and PR-positive invasive breast cancer (15, 17, 24). In contrast, BMI has been inversely associated with premenopausal breast cancer incidence. In a large pooled analysis of premenopausal women, the negative relationship between BMI and breast cancer risk was strongest in early adulthood (ages 18–24 years), and for hormone receptor-positive cancer (25). The negative relationship between breast cancer and premenopausal adiposity was not found with ER/PR-negative or triple negative (ER/PR negative, non-HER2 overexpressing) breast cancer in individuals over 24 years of age (25). It has been reported in a meta-analysis that a positive association exists between the presence of obesity in premenopausal women and the risk of triple negative breast cancer (TNBC) (26).

A large prospective cohort study following men and women over the course of 16 years found that being overweight or obese was associated with a higher risk of death from cancer (27). It has however, been noted that inconsistency exists regarding the relationship between obesity and increased cancer-specific mortality (15, 16, 28). The inconsistency between studies stems from multiple factors, including differences in study design and setting, timing of obesity measurements in relation to cancer diagnosis, cancer stage at diagnosis, presence of other risk factors such as smoking (29, 30), genetic variants, choice of treatment, and the effect of obesity on therapeutic dosing and adherence (15, 16, 28). Obesity has been consistently linked with breast cancer-specific mortality regardless of menopausal status or subtype across meta-analyses (31–33).

Although obesity has been defined using BMI in the majority of epidemiology studies, it does not always reflect metabolic health. High body fat levels correlated with fasting insulin, leptin, triglycerides and inflammatory markers (IL-6, C-reactive protein), and increased breast cancer risk in postmenopausal women with normal BMI (34). Waist circumference and waist-to-hip ratio are other measures that are used to define obesity (17, 35, 36). Waist circumference is one of the criteria that defines the metabolic syndrome. Abdominal obesity has a stronger correlation with insulin resistance than BMI or gluteofemoral (gynoid) obesity (37, 38). The metabolic syndrome, which is comprised of abdominal obesity, dyslipidemia, dysglycemia and hypertension, is a syndrome of insulin resistance (35). As discussed in subsequent sections, the metabolic dysfunction associated with insulin resistance may underlie the link between obesity and cancer.

### The Metabolic Syndrome and Insulin Resistance

Insulin resistance and hyperinsulinemia have been noted in individuals for many years preceding the diagnosis of diabetes (39). Obesity, specifically abdominal adiposity has been correlated with insulin resistance (40, 41). Insulin resistance is also considered to underlie the development of the metabolic syndrome. Endogenous hyperinsulinemia occurs to compensate for insulin resistance in order to maintain euglycemia. In clinical studies fasting insulin levels, or the fasting concentrations of C-peptide (a cleavage product of the insulin precursor that is released at equal concentrations to insulin) have been used to examine the links between hyperinsulinemia and cancer.

Studies have largely reported that the metabolic syndrome increases the risk for developing cancers such as, breast, colorectal, liver, bladder, endometrial and pancreatic cancer (42–45). In particular, it was observed that women with insulin resistance in comparision to insulin-sensitive women were at greater risk of developing breast cancer, regardless of BMI-defined obesity status (46). Circulating C-peptide (47), and insulin levels have been associated with increased breast cancer risk and cancer-specific mortality (48, 49), even after adjustment for adiposity (46, 50). Likewise, increased C-peptide or insulin levels have been linked with a greater risk of colorectal cancer, independent of adiposity (51, 52). Studies examining the timing of cancer diagnoses in individuals with diabetes found that the risk of cancer was higher in the period before diabetes diagnosis (53, 54), when insulin resistance and hyperinsulinemia is likely to be present. Overall, these observations support the hypothesis that hyperinsulinemia promotes cancer development and progression.

In addition to cancer incidence, insulin resistance has been associated with both an increased risk of all-cause mortality and cancer-specific mortality in postmenopausal women (55). Insulin resistance has been further identified as a factor mediating the relationship between race and poor breast cancer prognosis (56). In a recent cross-sectional study, self-identified Black women showed greater insulin resistance and poorer prognosis for breast cancer compared to White women (56). It is however, important to note that multiple factors including socio-economic status, environmental exposures, access to healthcare, tumor biology, genetic susceptibility and systemic metabolism can all potentially contibute to the racial disparities in cancer mortality (57).

### Type 2 Diabetes and Hyperglycemia

Obesity is commonly observed in individuals with type 2 diabetes and the rising cases of obesity have been proposed to explain the sharp increases in the prevalence and incidence of type 2 diabetes (58). Large cohort studies and meta-analyses have observed an increased risk of several types of cancer including breast (59), intrahepatic cholangiocarcinoma (60), colorectal cancer (59, 61) and pancreatic cancer (62) in individuals with type 2 diabetes. An umbrella review of meta-analyses, which also included an assessment of robustness and evidence of bias, showed that the incidence of breast, endometrial, colorectal cancers, and intrahepatic cholangiocarcinoma was greater in individuals with type 2 diabetes, compared to those without diabetes (63). A recent mendelian randomization study reported that a genetic predisposition to type 2 diabetes conveyed higher odds of cancer of the pancreas, kidney, uterus and cervix (64). For breast cancer, a meta-analysis across forty studies found that diabetes increased the risk of post-menopausal breast cancer by 16%, after adjustment for BMI (65). Subtypes of breast cancer that carry a poorer prognosis, including PR/HER2-negative breast cancers, TNBC, and the closely related basal-like breast cancer molecular subtype have also been reported to occur at greater frequency in women with diabetes than in those without diabetes (66, 67).

Diabetes has also been associated with increased cancer mortality (68, 69). One large cohort study followed individuals without a history of cancer at enrolment for 16 years, and found that diabetes was a significant predictor of mortality from liver, pancreatic, bladder and colon cancer in men, and pancreatic, colon, and breast cancer in women (68). The greater cancer mortality in individuals with diabetes was also reported in a large pooled analysis of 97 prospective studies with 820,900 participants and in many meta-analyses (61, 69–71). In particular, a meta-analysis examining survival outcomes for individuals with pre-existing diabetes and newly diagnosed cancer, found that those with diabetes had a 41% increased mortality compared to non-diabetic individuals (72). Although the evidence linking diabetes and all-cause mortality in individuals with cancer is strong, the evidence regarding cancer-specific mortality has been inconsistent, as individuals with diabetes have a greater mortality from non-cancer causes than those without diabetes (73). Diabetes may also have an impact on cancer treatment, as previously reported for breast cancer (74). However, a recent study which accounted for co-morbidities such as cardiovascular disease, found that cancer treatments were similar for patients with breast cancer regardless of diabetes status (75). Other possible factors relating to the increased cancer-specific mortality in individuals with diabetes, include presenting with advanced stage cancers at diagnosis (76), a higher risk of chemotherapy-related toxicity (77), as well as patient fragility resulting from chronic diabetes-associated complications (78).

Glycated hemoglobin (HbA1c) levels have been used as an indicator of glucose levels to examine possible associations between hyperglycemia and cancer in the UK biobank cohort, a large prospective population-based cohort study. Diabetes and HbA1c levels were observed to be positively linked with cancer risk across some organs, including liver and bladder (79). Another population-based cohort study, which also drew data from the UK biobank cohort, reported that with the exception of pancreatic cancer, HbA1c levels did not correlate with higher cancer risk, after adjustment for factors such as BMI, physical activity, alcohol consumption and ethnicity (80). A lack of association was also observed in a mendelian randomization study examining the relationship between breast and prostate cancer risk with glycemic traits (81).

### Dyslipidemia

Dyslipidemia is frequently associated with obesity and type 2 diabetes. Elevated levels of triglycerides and decreased high-density lipoprotein (HDL) are components of the metabolic syndrome and are often observed in conjunction with high levels of low-density lipoprotein (LDL) cholesterol, and small dense LDL (82). Triglycerides are transported in the circulation in the form of chylomicrons and very low density lipoprotein (VLDL), which is synthesized and secreted by the liver along with apolipoproteins (ApoB-100, ApoC-I, ApoC-II, ApoC-III, ApoE), which bind the non-polar lipids and aqueous plasma, facilitating the transport of non-polar lipids through the circulation (83). Dyslipidemia has been associated with increased cancer risk in some studies (84–87). Meta-analyses have examined dietary cholesterol intake, and found that high dietary intake of cholesterol increased the risk of esophageal cancer (88), pancreatic cancer (8% increased risk per 100 mg cholesterol/day) (89) and ovarian cancer (1% increased risk per 15 mg cholesterol/day) (90). In breast cancer, a dose-response analysis found that a non-linear relationship existed between dietary cholesterol and breast cancer, and was statistically significant when cholesterol intake was greater than 370 mg/day (91).

In a cohort of 3,278 adults from the Framingham Offspring study, individuals with high VLDL and low HDL levels had a greater incidence of cancer (92). Higher incidence of prostate and colon cancer in men, and breast cancer in women were reported in individuals with high cholesterol (≥ 240mg/dL), compared with those with cholesterol <160mg/dL in a large prospective study from Korea (93). A lower incidence of lung, liver and stomach cancers were found in individuals with high total cholesterol in this cohort (93). A meta-analysis of twelve prospective studies examining cancer risk irrespective of site found an inverse relationship between total cholesterol and cancer risk (94). It is important to note the distinction between total and HDL cholesterol, where low HDL is a component of the metabolic syndrome. In the context of breast cancer, an inverse association with cancer risk has been observed for HDL cholesterol (95). Menopausal status may impact upon the influence of cholesterol on breast cancer. For example, low HDL has been associated with increased risk of postmenopausal breast cancer in some studies (96, 97), but has been linked with premenopausal breast cancer in other studies (98). A few studies have looked at cancer incidences and mortality rates in individuals with familial hypercholesterolemia (FH). Though no difference was found in total cancer mortality rate (99), a strong association was found with death rates from pancreatic cancer (100). In contrast, lower incidences of smoking-related cancers in individuals with FH have been observed, which may be attributed to decreased smoking as a lifestyle modification (101).

The presence of many variables may explain the conflicting results that have been reported in epidemiological studies. In the setting of hepatocellular carcinoma or hepatic metastasis, impaired liver function may result in aberrant lipid synthesis, decreasing cholesterol levels (102). Similarly, individuals with pancreatic cancers and other gastrointestinal cancers may have decreased dietary absorption of lipids that would affect systemic lipid levels (103, 104). A relationship between circulating lipid levels and cancer risk may also be obscured by a decrease in lipid levels arising from the low nutritional intake associated with advanced cancer and cachexia (105). Increased cholesterol uptake by cells in some hematological malignancies has been described, and may also account for low lipid levels, increasing the difficulty in establishing a direct relationship between cholesterol and cancer risk (106). Furthermore, dyslipidemic individuals are often treated with lipid-lowering medications (107) as cardiovascular disease constitutes a co-morbidity of dyslipidemia. This in turn, might mask a direct association between cholesterol levels and cancer risk. Individuals with untreated high cholesterol levels are also at an increased risk of pre-mature cardiovascular disease-related mortality (107, 108). Therefore, cardiovascular disease may be a competing risk factor for mortality independent of cancer.

Cholesterol lowering 3-hydroxy-3-methylglutaryl-coenzyme A (HMG CoA) reductase inhibitors (“statins”) may have therapeutic value as anticancer agents. Although the evidence regarding statin use and its effect on cancer risk and mortality is not unanimous, many studies have reported a strong inverse relationship between advanced prostate cancer and longer duration of statin use (109–111). A large Danish population study found lower incidences of prostate and breast cancer in statin users compared with those who did not use statins (112). However, meta-analyses did not find a link between statin use and the occurrence of lung (113) and breast cancer (114). The majority of the studies examining statin use and breast cancer recurrence and prognosis suggest statins improve recurrence-free and cancer-specific survival (115–117).

## Mechanisms Underlying the Obesity–Cancer Relationship: Hyperglycemia and Insulin Signaling

### Insulin/Insulin-Like Growth Factor Signaling and Cancer

At a cellular level, activation of insulin/IGF signaling pathway has been hypothesized to contribute to tumor initiation and/or progression through tumor cell-specific mechanisms including the promotion of cell division, glucose metabolism (118) and epithelial-to-mesenchymal transition (EMT) (119). In addition to activating mitogenic and pro-tumorigenc metabolic pathways *via* the induction of endogenous hyperinsulinemia, insulin resistance might also contribute to tumor growth by other mechanisms including: modulating sex hormone bioavailability by decreasing sex hormone binding globulin; reducing of levels of certain circulating IGF binding proteins (IGFBP-1) resulting in free IGFs that activate the cell surface receptors; raising circulating triglycerides by enhancing hepatic lipid synthesis and decreasing clearance; increasing circulating and tissue free fatty acids from adipose tissue lipolysis, and altering expression of adipokines (**Figure 1**) (82).

Insulin, insulin-like growth factor-1 (IGF-1), and insulin-like growth factor-2 (IGF-2) are ligands for the transmembrane tyrosine kinase receptors, insulin receptor (IR), and IGF-1 receptor (IGF-1R), which have important roles in growth, development, cancer, and metabolic disease (120, 121). The IR is the preferential receptor for insulin and is comprised of two heterodimeric hemi-receptors, of which there are two isoforms, IR-A and IR-B (120). IR-A, in comparison with IR-B, displays an increased affinity for IGF-2 and as such, also acts a receptor for circulating and locally produced IGF-2 (122). Activation of the receptors *via* ligand binding results in trans auto-phosphorylation within the intracellular subunits of the receptors. This leads to the activation and recruitment of various substrates including the IR substrates (IRS) 1-4 and adaptor proteins. Pathways that are activated as a result of IR/IGF-1R activation include the Phosphatidylinositol 3-kinase (PI3K)/Akt/mechanistic target of rapamycin (mTOR) and Ras/extracellular signal-regulated kinase (ERK)1/2 pathways (121, 123).

The circulating levels of IGF-1 are positively associated with both increasing BMI up to 27 kg/m^2^ (124, 125) and increased risk of pre- and post-menopausal breast cancer (126, 127). The contribution of IGF-1 to the obesity-cancer link is not simple, as the positive relationship between IGF-1 levels and BMI exists only up to 27 kg/m^2^ and becomes negative thereafter (124), secondary to hyperinsulinemia inhibiting growth hormone secretion (128). In contrast to total circulating IGF-1, most of which exists in complexes with IGFBPs, the actual bioavailability of IGF-1 at a tissue level *in vivo* is difficult to determine (129), which complicates the contribution of IGF-1 to the relationship between obesity and cancer (124).

Rodent studies found that reduced circulating IGF-1 levels led to decreased tumor development (130) and that increased signaling through the IGF-1R signaling pathway promoted tumor growth (131, 132). The stimulation of an ER-positive breast cancer cell line (MCF7) with IGF-1 led to the identification of an IGF-1 gene signature, which was enriched for signaling pathways that are involved in mitogenesis such as ER, Ras/ERK1/2, and PI3K/Akt/mTOR. This IGF-1 gene signature was, in turn, associated with poorer survival (133). Increased activation of the IGF-1R signaling pathway led to reduced E-cadherin expression, and the potentiation of response to IGF-1R inhibition in the context of invasive lobular breast cancer, which is largely ER-positive (134). In the context of TNBC, low IGF-1R expression has been found and linked to worse overall survival (135). In Wnt-driven tumors, the inhibition of IGF-1R signaling led to increased mammary tumor development (136), potentially through a loss of protection from cellular stress and the development of a pro-metastatic tumor microenvironment (135). These observations suggest that the effect of IGF-1R signaling on breast cancer progression may be context-dependent.

### Insulin Signaling in Cancer

Relative to normal breast tissue, increased expression of the IR has been demonstrated in breast cancer tissue (137). The phosphorylation of the IR/IGF-1R has been noted across breast cancer sub-types, with 48.1% IR/IGF-1R phosphorylation in luminal, 64.3% in HER2-overexpressing, and 41.9% in TNBC cases examined (138). The IR has also been noted to be resistant to down-regulation in the setting of hyperinsulinemia (139). The stimulation of non-small cell lung, pancreatic and breast cancer cell lines, which express the IR, with insulin led to proliferation *in vitro* (140–142). Conversely proliferation was decreased with silencing of the IR (141–145). The tumor growth-promoting effect of endogenous hyperinsulinemia have also been shown in rodent models across several obesity-associated cancer types (118).

The stimulatory effect of endogenous hyperinsulinemia on breast cancer progression has been modeled through the use of a transgenic mouse model (MKR), in which a kinase-inactive form of the *IGF1R* is overexpressed in skeletal muscle under the muscle creatine kinase promoter (146). The female MKR mice display insulin resistance, as well as endogenous hyperinsulinemia in the absence of obesity (147). The stimulatory effect of hyperinsulinemia on both primary tumor growth and metastasis was demonstrated across a variety of breast cancer models employing different oncogenes (147–151) with tumors showing activation of the IR/IGF-1R and Akt (147). Tumors from the MKR mice, relative to wild-type mice, had increased levels of phosphorylated IR but not IGF-1R (152), thereby identifying the activation of the IR as a major contributor to hyperinsulinemia-associated tumor growth. The tumor promoting effects of hyperinsulinemia were ameliorated by either lowering circulating insulin levels with a β_3_-adrenergic agonist (153), or with inhibitors of the IR/IGF-1R, PI3K, and/or mTOR (147, 154, 155). These inhibitors while effective for reducing tumor growth, were shown to exacerbate the systemic metabolic abnormalities associated with insulin resistance: (hyperglycemia, hypertriglyceridemia, and hyperinsulinemia) in the MKR and wild-type mice (147, 154, 155). PI3K inhibitors contribute to hepatic glycogenolysis and reduced glucose disposal into adipose tissue, leading to greater secretion of insulin from the pancreas and hyperinsulinemia (123, 142). Insulin was observed to result in the re-activation of the PI3K signaling pathway and restoration of growth across a variey of cell lines including pancreatic and breast (142), ultimately reducing efficacy of PI3K inhibition in pre-clinical models. The use of PI3K inhibitors, in combination with insulin-lowering therapies, such as a ketogenic diet or sodium glucose co-transporter-2 (SGLT2) inhibition, resulted in sustained suppression of tumor growth in pre-clinical studies (142).

Insulin enhances glucose uptake in tissues such as muscle and adipose tissue by inducing the translocation of glucose transporter 4 (GLUT4) (82). The expression of the GLUT proteins, which comprise a family of 14 members, differs across tissues and tumor types, with the expression of GLUT1 and GLUT3 commonly being identified as elevated in cancer (156). Hyperglycemia is a defining feature of diabetes and has been postulated to mediate cancer progression through a variety of mechanisms including the promotion of DNA damage and accumulation of mutations, pro-tumorigenic post-translational protein modifications, acting as a metabolic substrate for cancer cells and by altering immune cell recruitment and activity (157–160). In particular, glucose is crucially involved in the Warburg effect which is commonly observed in cancer cells where the rate of glucose uptake is elevated and aerobic glycolysis occurs. The Warburg effect has been proposed to support cancer progression by allowing for the rapid generation of ATP and enhanced flux through biosynthetic pathways for cell proliferation and modulation of cell signaling and the tumor microenvironment, in part through acidification arising from lactate accumulation (161). Insulin has been suggested to influence tumor cell metabolism and anabolism by directing the utilization of glucose through PI3K-Akt signaling, leading to (1): enhanced glycolytic flux resulting in the generation of ATP (2); the promotion of aerobic glycolysis with the generation of lactate and the regeneration of NAD^+^ (3); increased production of ribose-5-phosphate, the precursor for purine and pyrimidine nucleotide synthesis, through the pentose phosphate pathway, and (4) increased lipid synthesis (162). In addition, insulin has also been shown to influence glucose metabolism through the regulation of cyclin D1-cyclin dependent kinase (Cdk) 4 activity (163), rendering hyperinsulinemia-associated tumor growth susceptible to CDK4 inhibitors in the context of liver cancer (164).

SGLT2 inhibitors are used to treat diabetes in clinical practice. SGLT2 inhibiton has also been examined in obesity-associated tumor studies in pre-clinical models of breast and colorectal cancer (165). The presence of hyperinsulinemia together with increased tumor proliferation and glucose uptake observed in obese rodents, was reduced with therapies with insulin-lowering effects, such as an SGLT2 inhibitor or a liver-specific mitochondrial uncoupler of oxidative phosphorylation. Treatment with insulin abrogated the tumor-suppressive effect associated with these therapies (165, 166). These observations taken together, suggest that insulin promotes tumor growth which is in turn, associated with increased glucose uptake, in some cancers. Interestingly, a positive association between glucose uptake into tumors and BMI has been found in the context of breast cancer, while glucose uptake in non-small cell lung cancer was inversely associated with BMI (167). In addition to increased tumor growth, the presence of elevated endogenous insulin levels and IR signaling have also been postulated to encourage metastasis through the promotion of EMT in both human (151) and mouse tumor models (149, 168) in the context of breast cancer and prostate cancer (169).

## Mechanisms Underlying the Obesity–Cancer Relationship: The Contribution of Lipids

### Cholesterol Uptake

In non-cancer cells, cholesterol and related sterols contribute to essential physiological functions and are crucial components in the membranes of eukaryotic cells, reducing permeability and influencing protein assembly (170). Cholesterol is also an essential molecule for the synthesis of other sterols, including steroid hormones and oxysterols (171). Cholesterol can be absorbed from extraneous sources, or synthesized intracellularly by the mevalonate pathway utilizing the rate-limiting enzyme, HMG CoA reductase. In addition to synthesizing cholesterol, the mevalonate pathway also gives rise to non-sterol isoprenoids, such as dolichol, coenzyme Q, farnesyl-pyrophosphate (FPP) and geranylgeranyl-pyrophosphate (GGPP) (172). Isoprenoids can prenylate many molecules important for carcinogenesis such as Ras GTPases that can lead to proliferation, migration, and metastasis (173).

Free cholesterol in cells is maintained at a constant level by homeostatic processes involving sensors like sterol regulatory element binding protein (SREBP) and liver X receptor (LXR). Nuclear translocation of SREBP leads to the enhanced expression of enzymes such as HMG CoA reductase, and the LDL receptor (LDLR), which contributes to increased exogenous lipid uptake (174). Enhanced activation of the SREBPs has been observed in cancers such as prostate (175), and breast cancer (176). Activation of the SREBP–mevalonate pathway sustained the proliferation and self-renewal of breast cancer cells in the setting of p53 mutations (177). The nuclear translocation of SREBP in the context of cancer is mediated by multiple factors such as the loss of the tumor suppressor p53, low pH of the tumor microenvironment, proinflammatory cytokines such as TNFα, and endoplasmic reticulum stress (178). LXRs are nuclear receptors that also modulate intracellular cholesterol levels by up-regulating the transcription of efflux protein such as ATP binding cassette subfamily A member 1 (ABCA1) and ATP binding cassette subfamily G member 1 (ABCG1). Upregulation of these efflux proteins by LXR agonists have induced apoptosis in prostate and breast cancer cell lines (179).

The influence of elevated circulating cholesterol on cancer growth has been modeled using rodents. Adiponectin knockout mice which have glucose intolerance, insulin resistance, and hyperlipidemia were observed to develop larger transgenic polyoma virus middle T antigen (PyMT) mammary tumors than control mice, and the effect was enhanced by high fat diet feeding (180). Similarly, syngeneic breast cancers in ApoE^-/-^, LDLR^-/-^, and APOE3^+/+^ mice, which are all models of hyperlipidemia, demonstrated increased growth compared to controls; ER-negative tumors in ApoE^-/-^and LDLR^-/-^ mice, and ER-positive tumors in APOE3^+/+^ (181–183). The tumor growth-promoting effect of hyperlipidemia has also been recapitulated in human breast cancer xenografts in immunodeficient mice models of hyperlipidemia (182).

An increased uptake of cholesterol into cancer cells has been observed (184–186) with LDLR expression being upregulated in certain breast cancer cell lines (182, 187). The scavenger receptor, SR-B1, is another means by which tumor cells may take up cholesterol. An increased expression of SR-B1 in breast and prostate cancer cells has been associated with increased cell proliferation and tumor growth *in vivo* (188, 189). Higher LDL metabolism has been observed in gynecological cancer cell lines compared non-neoplastic cells (190). The tumor cell expression of LDLR plays a crucial in the uptake of circulating LDL and the growth of pancreatic adenocarcinoma and prostate cancer (184, 191). Silencing the LDLR in breast cancers reduced tumor growth, particularly in the setting of high circulating LDL (182). Furthermore, in human breast cancers a high expression of LDLR was associated with decreased recurrence-free survival in patients who have received systemic therapy (182). Interestingly, LDL uptake through the LDLR on cancer cells is being studied as a mechanism of targeted drug delivery to tumors, using lipidic emulsions. These molecules were reported to localize heavily in human breast cancer cells that were removed during surgery compared with normal cells (192). LDLR could therefore, be a potential drug target. Taken together these observations suggest that the increased cholesterol uptake and metabolism by cancer cells may support rapid cell division and growth.

Stored cholesterol in the form of cholesteryl esters (CE) may contribute to proliferation and aggressiveness of breast (193), prostate, and colon cancer (194), as well as leukemia (195). Increased activity of Acetyl-coenzyme acetyltransferase-1 (ACAT1), an enzyme that can catalyze cholesterol esterification (196) as well as lipase activity (197) has also been seen in cancer cells, suggesting that CE may allow cancer cells to store and quickly access energy when needed. Cholesterol is also located in lipid rafts, which are crucial for cell signaling, adhesion and migration of cancer cells. The depletion of cholesterol from lipid rafts using methyl-β-cyclodextrin (MβCD) resulted in the disruption of lipid rafts and increased apoptosis of breast cancer cells (198). Although high intracellular cholesterol levels appear to be conducive for cancer cell growth and survival, it has also been noted that low concentrations in some cases, facilitate metastasis by enhancing membrane fluidity and the consequent development of a migratory phenotype (199).

Cholesterol lowering statins may exert anti-cancer effects by lowering circulating cholesterol levels or targeting the mevalonate pathway in cancer cells leading to decreased cholesterol synthesis (200). Statins can either be hydrophilic, which are predominantly taken up by the liver, or lipophilic, which can be taken up by all cells by passive diffusion (201). Lipophilic statins have been found to reduce cancer risk suggesting that their beneficial effect may be by acting on tumor cells directly (202). Statins also confer pleiotropic benefits, such as improved endothelial function, antioxidant properties and anti-inflammatory properties (203). Their anti-inflammatory role is suggested by a reduction in C-reactive protein (204) and may occur in part, by lowering levels of pro-inflammatory cytokines such as TNFα and IFNγ (205). These effects are beneficial in treating cardiovascular disease. In breast cancer models, statins blocked tumor growth by: 1) inducing cell cycle arrest by cyclin D1-CDK4; 2) decreasing cell proliferation through inhibiting prenylation and activation Rho/Ras family of proteins; 3) modulating pro- and anti-apoptotic proteins such as Bcl2 and Bax, and by inducing reactive oxygen species (ROS) (202), and 4) contributing to oxidative stress by inhibiting coenzyme Q synthesis (206). Statins have also been shown to suppress angiogenesis, invasion and metastasis through reducing the expression of MMP2, MMP9 and VEGF (202).

### Oxysterols: 27-Hydroxycholesterol

27-Hydroxycholesterol (27-HC) is the most abundant circulating oxysterol in humans (207). 27-HC is produced when cholesterol is hydroxylated by cytochrome P450 27A1 (CYP27A1) to yield 27-HC in both the liver and extrahepatic tissues. Levels of 27-HC are decreased by cytochrome P450 family 7 subfamily B member 1 (CYP7B1), which metabolizes it to produce an intermediate for bile acid synthesis in the liver (208, 209). 27-HC was the first discovered endogenous selective estrogen receptor modulator (SERM) (210). Though 27-HC binds to both ERα and ERβ, it binds to ERβ with >100 more affinity than ERα (211). In the breast it shows partial agonist activity to ERα, but has lower efficacy than 17β-estradiol (210, 212). However, in other tissues such as cardiovascular or bone, it acts as an antagonist and can attenuate the protective effects of estrogen (209, 213, 214). It is also a LXR agonist and has the ability to bind to both LXRα and LXRβ to modulate LXR dependent genes that are involved in lipid homeostasis such as ABCA1, ABCG1, and SREBP-1c. The expression of LXR-related genes can be modulated with cholesterol loading, suggesting that 27-HC may act as a cholesterol sensor (208).

Plasma levels of 27-HC have been shown to correlate with circulating cholesterol levels (215) and has been hypothesized to explain the link between hypercholesterolemia and cancer (216, 217). 27-HC content in normal breast tissue from individuals with cancer was increased compared with cancer-free controls, and was observed to be further elevated in tumors (218). 27-HC was also observed to be elevated in exosomes from ER-positive but not ER-negative breast cancer or other non-cancerous cell lines (219). Decreased expression of the enzyme, CYP7B1, has been linked to poorer cancer prognosis (218). Silencing of *CYP7B1* can occur epigenetically by hypermethylation of the gene (220). In contrast, the relationship between the expression of CYP27A1 in tumors and cancer prognosis has been inconsistent. Intratumoral expression of CYP27A1 was linked to high grade cancer cells and ER-positive breast tumors (183). However, in three independent cohorts of breast cancer patients, elevated levels of CYP27A1 correlated with longer recurrence-free survival in women with ER-positive breast cancer who were less than 50 years of age (221). In a recent evaluation of a cohort from the European Investigation into Cancer and Nutrition (EPIC) study, no association between circulating 27-HC levels and premenopausal breast cancer risk was found, and an inverse correlation was reported in postmenopausal women (222, 223). These results suggest that the estrogen levels may influence the association between 27-HC and breast cancer risk and survival (224).

27-HC stimulates the proliferation of ER-positive breast cancer cells by increasing cyclin D activity (212) and by reducing p53 transcriptional activity by facilitating its interaction with MDM2 (225). The growth-promoting effect of 27-HC has also been re-capitulated *in vivo*, where ER-positive tumor xenografts displayed accelerated growth in the presence of 27-HC (183). Increased levels of 27-HC were also found to promote metastasis. Inhibition of Cyp27a1 with a small molecule inhibitor, or by genetic ablation resulted in reduced lung metastasis (226). 27-HC induced EMT in MCF7 cells by reducing the expression of E-cadherin and β-catenin and increasing the expression of MMP9 (227, 228). In both MCF7 and MDA-MB-231 breast cancer cell lines, 27-HC increased ROS production that led to increased migration of the cells *via* STAT3/VEGF signaling (229, 230). In addition to affecting tumor cells directly, 27-HC induced endothelial to mesenchymal transition in endothelial cell lines *via* STAT3 signaling and aided breast cancer cell migration (231). It also influenced the tumor immune microenvironment in the lung, where 27-HC increased recruitment of polymorphonuclear neutrophils (PMNs) and γδ T-cells, which suppresed the recruitment of anti-tumorigenic effector T-cells (226).

**Figure 2:**
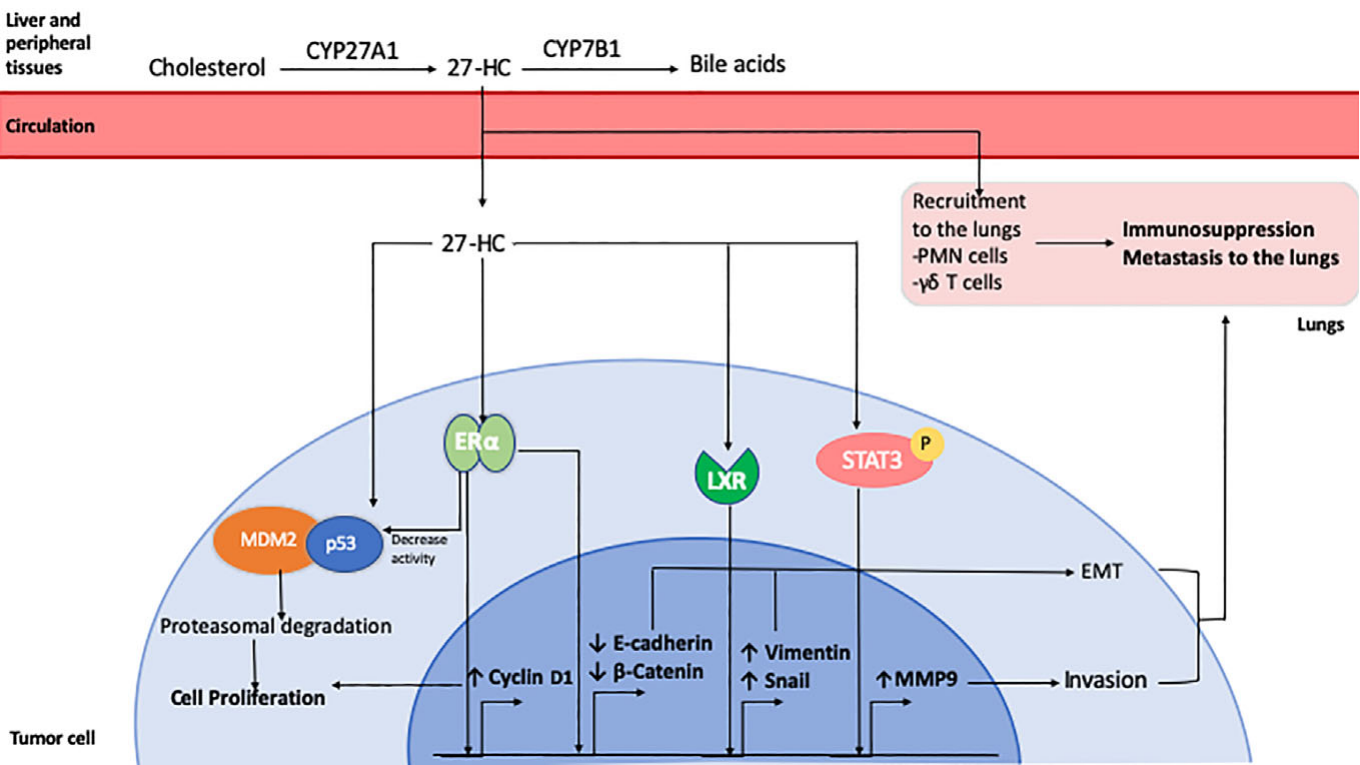
Role of 27-Hydroxycholesterol in breast cancer. 27-HC is synthesized from cholesterol by CYP27A1 in the liver and other peripheral tissues. It can be further metabolized for bile acid synthesis by CYP7B1. 27-HC can be taken up by tumor cells from the circulation where it exerts ERα agonistic activity, inducing the expression of Cyclin D1 which leads to cell cycle progression and proliferation. It can also enhance the association of tumor suppressor p53 protein with MDM2 leading to its degradation. 27-HC has also been shown to promote EMT by reducing E-Cadherin and β-Catenin expression and by inducing an LXR-mediated increase in Snail and Vimentin expression. It can also activate Stat3 signaling, resulting in increased expression of MMP9 and cell invasion. 27-HC in the lungs can promote the recruitment of polymorphonuclear cells and T cells which favors the development of an immunosuppressive microenvironment that facilitates metastatic seeding and growth of breast cancer cells.

Since the discovery of the role of 27-HC in breast cancer progression, new research shows that its effect is not restricted to breast cancer. 27-HC increased the proliferation of lung, endometrial and melanoma cancer cell lines (232–234). In contrast, 27-HC suppressed the proliferation of gastric, and colon cancer cell lines. (236–237). In DU145 prostate cancer cells, 27HC inhibited proliferation by disrupting lipid rafts (235), but increased the proliferation of LNCaP and PC3 cells via an ERβ-mediated pathway (238). However, lower CYP27A1 transcript levels were associated with shorter disease-free survival and higher tumor grade in patient samples (239). Therefore, 27-HC may mediate some of the pro-tumorigenic effects of cholesterol through direct effects on tumor cells, and indirectly by its effects on the tumor microenvironment (**Figure 2**). However, a greater understanding of its tumor promoting and inhibiting properties is needed, in addition to understanding whether circulating or locally produced 27-HC has a greater influence on tumor growth and metastasis.

### Fatty Acid Transfer

Insulin resistance in adipose tissue leads to lipolysis and the release of fatty acids may also support tumor growth (240). Fatty acid uptake and metabolism has been reported to promote tumor cell proliferation, survival, invasion, and tumor-initiating capacity (241–247). The expression of fatty acid translocase, CD36, was identified as a characteristic of metastasis-initiating cells (243). CD36 expression was induced in cancer cells co-cultured with adipocytes in the context of ovarian cancer, leading to increased tumor cell fatty acid uptake, invasion and proliferation (248). Fatty acid transfer and the expression of genes associated with lipid metabolism and inflammation were enhanced when tumor cells were cultured with hypertrophied adipocytes isolated under obesity-associated conditions compared to adipocytes from non-obese conditions (249, 250).

The enhanced provision of fatty acids in the context of adipose tissue lipolysis may also influence immune cells. In natural killer (NK) cells, obesity was associated with enhanced expression of lipid transport genes, such as Ldlr, Cd36, and fatty acid binding proteins (Fabp), along with decreased mTOR-associated glycolysis, which was related to activation of PPAR_α/δ_ signaling. These metabolic changes in NK cells resulted in reduced cytotoxic activity (251). While not studied directly in the context of obesity, macrophages, myeloid-derived suppressor cells (MDSCs) and regulatory T-cells (Treg), have also been shown to up-regulate lipid transport receptors and increase lipid uptake in lipid-enriched tumor microenvironments, leading to pro-tumorigenic effects (252–254). In particular, CD36 was shown to mediate lipid accumulation and fatty acid oxidation in tumor-associated macrophages, resulting in a pro-tumorigenic gene expression profile (253). In MDSCs, the expression of lipid transporters including Cd36, Vldlr, Ldlr, and Fabp were induced by cytokines, through STAT3/5 signaling, leading to enhanced immunosuppressive capacity (252). Tregs are associated with the suppression of anti-tumor immune responses (255). Tregs were shown to utilize fatty acid oxidation during their development and possess an enhanced capacity for fatty acid oxidation through the expression of the Treg-specific transcription factor, Foxp3, which has been proposed to afford them protection from fatty acid toxicity (256, 257). Tregs situated in tumors increased both the expression of the fatty acid receptor, CD36, and lipid uptake, leading to a PPAR_β_-driven increase in capacity to survive in lactic acid-rich tumor microenvironments (254). Ablation or neutralization of CD36 led to the loss of suppressive function and reduced tumor growth in mice, demonstrating the importance of fatty acid uptake by Tregs (254).

Effector T-cell numbers were observed to be reduced in tumors of mice with diet-induced obesity. Inhibiting fatty acid oxidation or the ablation of STAT3, increased effector T-cell glycolysis and T-cell numbers, resulting in decreased tumor growth (258). However, fatty acid oxidation has been found to be important for effector T-cell function under hypoxic and hypoglycemic conditions in melanoma models (259). Enhancing fatty acid oxidation concurrently with glycolysis with a PGC-1α/PPAR agonist improved responses to immunotherapy in models of colon cancer and skin sarcoma (260).

A lipid-enriched environment may therefore, promote tumor growth by reducing NK cell cytotoxic function and effector T cell numbers, and enhancing the accumulation and immunosuppressive function of macrophages, MDSCs, and Tregs.

## Mechanisms Underlying the Obesity–Cancer Relationship: Obesity-Associated Adipose Tissue Expansion

### Adipose Stromal Cells

Adipose tissue is composed of a variety of cell types in addition to adipocytes, such as adipocyte progenitor cells, fibroblasts, endothelial cells, and immune cells, that contribute to its behavior and diverse adipocytokine secretion profile (261, 262). The expansion of adipose tissue with the development of obesity occurs due to a combination of adipogenesis and lipogenesis, processes that are regulated by insulin/IR signaling (263–265).

The obese phenotype has been found to promote breast cancer growth through an enhanced provision of adipose tissue progenitor cells (also known as adipose stromal cells), which express CD34 and can be differentiated into multiple cell types such as chondrocytes, osteocytes and adipocytes (266). These adipose tissue progenitor cells are detectable in the circulation of both rodents (267) and humans and are present at increased levels with obesity (268, 269). Co-injection experiments involving adipose tissue-derived CD34^+^ progenitor cells and tumor cells resulted in increased tumor growth and metastasis in mice compared to injections of tumor cells alone, demonstrating the potential for adipose tissue progenitor cells to contribute to the tumor vasculature and promote tumor growth (270). In rodent models, diet-induced obesity resulted in both an elevation in the levels of adipose stromal cells in the circulation and increased engraftment of these stromal cells in tumors, with an increased number of actively proliferating tumor cells positioned closer in proximity to the adipose tissue-derived structures (267). C-X-C motif chemokine ligand 1 (CXCL1) signaling has been shown to recruit C-X-C motif chemokine receptor (CXCR)1/2 expressing progenitor cells into prostate cancers (266, 267, 271). The recruitment of adipose stromal cells has also been associated with increased levels of GM-CSF and MMP9 levels in tumors, which promote the development of a tumor-supportive microenvironment (272). In addition to contributing to the development of the tumor microenvironment and vasculature, the recruitment of adipose stromal cells has also been linked to the promotion of EMT and chemo-resistance of prostate cancer cells, where the ablation of adipose stromal cells was shown to augment the efficacy of chemotherapy (273).

### Obesity-Associated Extracellular Matrix Deposition

The expansion of adipose tissue also involves the synthesis and deposition of extracellular matrix proteins (fibrosis) (274), which has been proposed to contribute to the development of dysfunctional adipose tissue in obesity and cancer (275). Compared to lean individuals, breast tissue from obese individuals contain a greater number of myofibroblasts, a cell type implicated in fibrotic remodeling, and stiffer extracellular matrices (276). These changes resulted in increased tumor cell growth *in vitro* (276).

Extracellular matrix (ECM) proteins such as collagen VI have been implicated in the link between obesity and cancer. Collagen VI expression in human subcutaneous adipose tissue was observed to be elevated in obese individuals compared to lean individuals, and has been associated with decreased insulin sensitivity and adipose tissue inflammation (277). Collagen VI, which is secreted by adipocytes, has been shown to stimulate cancer growth, while collagen VI-null mice had reduced tumor growth in the context of breast cancer (278, 279).

The expression of endotrophin (ETP), a collagen VI fragment, is higher in the adipose tissue of obese mice and has been implicated in both obesity-associated inflammation and metabolic dysfunction (280). ETP was also present at greater levels in the circulation of women with breast cancer relative to cancer-free women (281). ETP promoted EMT and chemo-resistance in human breast cancer cell lines (281) and enhanced primary and metastatic tumor growth in mice (279). Neutralization of endotrophin with a humanized anti-ETP antibody reduced tumor growth in a preclinical model (281). Taken together, these observations illustrate how excess ECM protein deposition, which is linked to obesity-associated adipose tissue inflammation and metabolic health, promotes tumor growth.

### Adipose Tissue Macrophages

Macrophages are an essential part of the tumor microenvironment with the ability to influence various processes that are involved in cancer development and progression, such as the promotion of tumor cell migration, angiogenesis, and regulation of the tumor immune response (282–284). Increased levels of the cytokines, C-C motif chemokine ligand 2 (CCL2) and Interleukin (IL)-1β, in adipose tissue from obese mice induced macrophage secretion of CXCL12, a cytokine that promotes angiogenesis (283). Accumulation of tumor-infiltrating macrophages with activated NOD-like receptor C4 (NLRC4) inflammasomes in the obese tumor microenvironment has been linked to increased IL-1β activation (284). IL-1β has been shown to promote tumor angiogenesis by stimulating adipocyte secretion of the proangiogenic cytokines, VEGF-A and ANGPTL4 (284, 285). Changes in adipose tissue ECM proteins associated with obesity skewed macrophage function towards a tumor-associated macrophage-like phenotype (286). The role of macrophages in obesity-associated cancer growth is however, complex. In a pre-neoplastic model, the depletion of macrophages in diet-induced obese mice led to greater numbers of mammary epithelial cells exhibiting DNA damage and increased mammary epithelial cell progenitor activity, suggesting that macrophages potentially play a modulatory role in the early stages of cancer development (287).

The accumulation of crown-like structures (CLS), which are dying (necrotic-like) adipocytes surrounded by macrophages, has been used as an indicator of inflammation and macrophage infiltration in adipose tissue (288). Breast adipose tissues from women with the metabolic syndrome have increased numbers of CLS. This association has been observed in both obese and non-obese women (BMI < 25 kg/m^2^), suggesting a link between insulin resistance and breast adipose tissue inflammation (289, 290). Breast adipose tissue CLS have been correlated with increased breast cancer risk (291–294), and decreased relapse-free survival (289). The relationship between the increased CLS formation and BMI (295) has been reported in African American, Hispanic/Latina, Asian, and White populations (290, 296). In prostate cancer, inflammation in periprostatic white adipose tissue was associated with higher grade, suggesting adipose tissue inflammation may have tumor-promoting effects in a number of cancers (297).

The accumulation of CLS in breast adipose tissue has been linked to increased ER-positive breast cancer risk through the increased secretion of aromatase inducers (TNF α, IL-1β and prostaglandin E2) by macrophages (298), and higher circulating concentrations of IL-6, leading to greater aromatase expression in pre-adipocytes (299). Aromatase is the enzyme that converts androgens to estrogens (292). In postmenopausal women, greater levels of breast adipose tissue inflammation and aromatase expression have been observed, potentially explaining the high incidence of ER-positive breast cancer compared to other breast cancer subtypes after menopause (300, 301).

### The Role of Leptin

In addition to the obesity-associated influence on macrophage behavior, the altered anti-tumor immune response observed in obesity has been proposed to be mediated by other mechanisms, some of which are linked to leptin.

Leptin is an adipokine that affects various physiological processes, including metabolism, reproduction and body weight regulation (302). Due to the development of leptin resistance, the circulating levels of leptin increase with BMI in both rodents and humans (303). Leptin has largely been reported to have a tumor growth-promoting influence in rodent models of breast cancer (304). Leptin-deficient obese mice had decreased mammary tumor growth relative to wild type in syngeneic models, and leptin receptor (ObR)-deficient mice (with high leptin levels), showed greater mammary tumor growth with syngeneic cancer models (305). Of note, tumor growth in the A-ZIP/F-1 mice, which are fatless and do not have detectable levels of leptin, was enhanced compared to wild-type mice. The A-ZIP/F-1 mice had increased levels of pro-inflammatory cytokines, and were also hyperinsulinemic and hyperglycemic, highlighting the pro-tumorigenic influence of these conditions even in the absence of leptin (306). In addition to stimulating tumor cell proliferation and invasion (307–309), leptin signaling was also reported to promote EMT and the generation of cancer stem cells (310, 311). Leptin can also modulate the behavior of immune cells.

The ObR is present in multiple types of leukocytes such as bone marrow CD34^+^ hematopoietic precursor cells, monocytes, macrophages, T- and B-cells (312), as well as a sub-population of NK cells (313). Leptin stimulation has been reported to affect the function of these immune cells, although the duration of exposure to leptin results in different effects. In human NK cells, short-term exposure to leptin led to increased anti-tumor activity and long-term exposure resulted in impaired anti-tumor function (313), suggesting that NK cell activity in the context of obesity might be impaired in the setting of chronic hyperleptinemia. Blocking the ObR decreased the number of circulating MDSCs (314).

Studies have found that the response to immunotherapies such as anti-CTLA-4 monoclonal antibodies (mAb), or recombinant adenoviral-encoded TNF-related apoptosis-inducing ligand Ad(TRAIL) combined with the TLR agonist, CpG, was impaired in obese mice compared to lean mice (315). The neutralization of leptin in obese mice potentiated the response to AdTrail/CpG immunotherapy leading to increased dendritic cell function and intratumoral T-cell accumulation, resulting in decreased tumor growth (315). In contrast to the response to immunotherapies involving AdTrail/CpG or anti-CTLA-4 mAb, the response to anti-PD-1 mAb was augmented in the context of obesity preclinical models (316). The favorable response was shown to be leptin-mediated. Exposure to chronically elevated levels of leptin in the context of obesity led to the development of an exhausted phenotype in T-cells, concomitant with an increased expression of PD-1. In contrast, PD-1 expression was not increased in T-cells from leptin-deficient or ObR-deficient mice, providing a mechanistic explanation for how response to this form of immunotherapy is potentially enhanced in the setting of obesity (316).

Consistent with the pre-clinical studies, an obesity paradox has been described for treatment responses to immune check-point inhibitors where high BMI has been associated with greater treatment efficacy (316–319). This effect has been described for cancers such as melanoma (319) and non-small cell lung cancer (318). It has also been noted that obesity was associated with the development of immune-related adverse effects (320–322), which in turn, was associated with better response to therapy (323, 324).

## Discussion

The obesity-cancer relationship is mediated by multiple mechanisms which are inter-related. Dysfunctional adipose tissue, characterized by increased inflammation and fibrosis plays a major role in driving obesity-associated cancer progression. Evidence from both preclinical studies and cross-sectional human clinical studies (261, 262, 325, 326) suggest that there is substantial interaction between adipose tissue and tumor cells, contributing to the obesity-associated promotion of tumor growth. In addition to a direct influence on tumor cells, the secretion of cytokines and fatty acids from adipose tissue may also promote tumor progression by modulating the accumulation and function of tumor-infiltrating leukocytes. As discussed, factors comprising metabolic health, such as dyslipidemia, insulin resistance and its corollary, hyperinsulinemia, promote cancer progression, largely through their effects on tumor cell growth and invasiveness. Further understanding how dyslipidemia and hyperinsulinemia might influence the development of the tumor microenvironment is an important area requiring futher investigation.

Therapeutic approaches to target different aspects of the obesity-cancer link have been reviewed recently (119, 261). These include targeting adipose tissue inflammation, reducing circulating insulin levels through dietary, pharmacological or surgical means (119, 261, 327), and decreasing circulating and tumor cell lipid synthesis, uptake and metabolism (244, 328). Given the complex relationship between systemic metabolic disease and the tumor immune microenvironment, it seems important that we not only consider how the anti-tumor immune response can be augmented with therapies targeting systemic metabolism, but also consider systemic metabolic conditions as variables in determining response to immune-based therapies in clinical trials.

In conclusion, characterizing and understanding the cross-talk between adipose tissue and tumors, the effect of obesity on immune cell function, and the effect of metabolic health on cancer progression will be key to improving responses to current therapies, and developing novel therapies that target systemic metabolic disease and cancer.

## Author Contributions

TS and AE drafted sections of the manuscript and edited it for cohesion. DL and EG conceived the project, reviewed and edited the manuscript. All authors contributed to the article and approved the submitted version.

## Funding

EG received research support from the National Institutes of Health/National Cancer Institute K08CA190770, Alkeon Capital, the Department of Medicine and the Tisch Cancer Institute at Mount Sinai. DL received research support from National Institutes of Health/National Cancer Institute R01CA200553 and R01CA128799.

## Conflict of Interest

TS and AE declare no conflicts of interest. EG declares the following potential conflicting interests: EG has served on an advisory board for Novartis Pharmaceuticals and as consultant for Seattle Genetics. DR declares the following conflicting interests: DR has served on advisory boards for Mannkind and AstraZeneca.
